# A novel auto-fluorescent porphyrin-lipid nanoparticle strategy for CTNNB1 gene silencing in hepatocellular carcinoma

**DOI:** 10.3389/fonc.2026.1779803

**Published:** 2026-05-08

**Authors:** Jeffrey C. To, Alyssa Apilan, Elisa Pasini, Yulin Mo, Juan Chen, Anita Bakrania, Jiachuan Bu, Anni Pan, Oyedele Adeyi, Arndt Vogel, Gang Zheng, Mamatha Bhat

**Affiliations:** 1 Ajmera Transplant Centre, University Health Network, Toronto, ON, Canada; 2 Princess Margaret Cancer Centre, University Health Network, Toronto, ON, Canada; 3 Division of Gastroenterology and Hepatology, Toronto General Hospital, Health Network, Toronto, ON, Canada; 4 Department of Pathology and University of Minnesota Medical Center, University of Minnesota, Minneapolis, MN, United States; 5 Anatomic Pathology, The University of Alabama at Birmingham, Alabama, AL, United States; 6 Department of Gastroenterology, Hepatology, Infectious Diseases and Endocrinology, Hannover Medical School, Hannover, Germany; 7 Department of Medical Biophysics, University of Toronto, Toronto, ON, Canada; 8 Department of Medicine, University of Toronto., Toronto, ON, Canada; 9 Division of Gastroenterology & Hepatology, University of Toronto, Toronto, ON, Canada

**Keywords:** CTNNB1, hepatocellular carcinoma (HCC), lipid nanoparticles (LNPs), porphyrin-lipid nanoparticles, RNA interference, targeted gene therapy, β-catenin

## Abstract

**Background:**

Hepatocellular carcinoma (HCC) remains a high-fatality cancer with limited effective therapies. CTNNB1 mutations, frequently observed in HCC, are associated with poor prognosis and immune evasion. CTNNB1 has long been considered an undruggable target due to its structural characteristics and extensive protein interactions. Porphyrin lipid nanoparticles (porphyrin-LNPs) are capable of targeting liver tumor cells, and their inherent autofluorescence allows evaluation of nanoparticle biodistribution in the liver. Our goal was to formulate a porphyrin-LNP encapsulating CTNNB1-targeting siRNA, as a novel strategy to target β-catenin-driven HCCs.

**Methods:**

We developed porphyrin-LNPs for systemic delivery of CTNNB1-targeting siRNA. Porphyrin-LNPs were synthesized via microfluidic rapid mixing and characterized by cryo-TEM and dynamic light scattering. Their delivery efficacy was validated in HCC cell lines (Hep3B, HepG2), and their therapeutic potential was evaluated in a murine model of CTNNB1/KRAS-driven HCC.

**Results:**

Porphyrin-LNPs showed high encapsulation efficiency (97%) and effective siRNA delivery *in vitro*. Treatment with porphyrin-LNP-si-CTNNB1 resulted in approximately 90% downregulation of *CTNNB1* expression (*p* < 0.0001 in Hep3B at 50nM; *p* < 0.0001 in HepG2 at 10nM) and significantly reduced clonogenic survival in both Hep3B (50% reduction at 50nM, *p* < 0.0001) and HepG2 (75% reduction at 10nM, *p* < 0.001) cell lines. *In vivo*, porphyrin-LNP-si-CTNNB1 significantly reduced tumor burden by approximately 67% (*p* < 0.0001), liver-to-body weight ratio by 50% (*p* < 0.0001), histological tumor grade, and β-catenin expression in CTNNB1/KRAS-driven HCC mice by approximately 58% (*p* < 0.0001).

**Conclusions:**

This study demonstrated that porphyrin-LNPs can effectively deliver siRNA to silence CTNNB1, an oncogene that has so far been undruggable in HCC. Future studies should explore biodistribution, immune modulation, and combination strategies to enhance clinical translatability of CTNNB1-targeted RNA interference in HCC.

## Graphical Abstract

**Figure:**
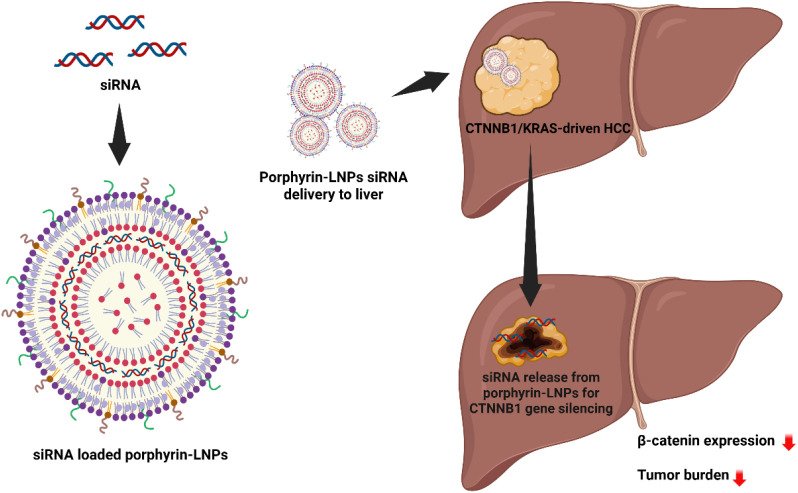

## Introduction

Hepatocellular carcinoma (HCC) is a high-fatality cancer with a dismal 18% 5-year survival rate (1, 2). This tumor poses a formidable challenge due to its grim prognosis, particularly in advanced stages (3–5). Current therapies yield response rates of only 20-30%, with up to 40% of patients failing to achieve adequate tumor control (3, 6, 7).

Cirrhosis is present in up to 90% of patients diagnosed with the disease, including those with metabolic dysfunction-associated steatohepatitis (MASH), viral hepatitis, and alcohol-related liver disease (8, 9). Cirrhosis and impaired liver function may significantly limit therapeutic options and impair hepatic drug metabolism (10–13). Although systemic treatments for advanced HCC have progressed markedly—particularly with ICIbased combination regimens—longterm survival or cure remains uncommon. Moreover, despite the approval of several tyrosine kinase inhibitors, validated biomarkers to guide systemic therapy selection have not been established. Unlike cholangiocarcinoma, many genetic alterations frequently observed in HCC are still deemed undruggable. Therefore, there is an urgent need to develop novel strategies to target oncogenic drivers in this devastating disease.

To circumvent the shortcomings of traditional treatments, nanoparticle-mediated siRNA therapy presents a promising alternative for HCC patients. Nanoparticles can be engineered to specifically deliver siRNA to cancer cells with high precision, thereby minimizing off-target effects and reducing potential damage to healthy tissues (14). This approach also offers the advantage of preferentially targeting genes of interest, including undruggable targets such as CTNNB1, a commonly mutated gene in HCC (14). To date, only two studies have reported the use of lipid nanoparticles targeting CTNNB1 in HCC (15, 16), highlighting the potential of nanomedicine for HCC treatment. Currently, ALN-BCAT (Alnylam Pharmaceuticals) comprises a chemically modified siRNA encapsulated in a lipid nanoparticle formulation, which achieves robust and highly specific reductions in CTNNB1 activity and is currently being evaluated in a Phase 1 clinical trial (NCT06600321). Nevertheless, systematic evaluations of LNP–siRNA delivery and biodistribution across different organs, and their relative activity in HCC tumors compared with normal hepatocytes, remain limited.

Lipid nanoparticles (LNPs) are modular delivery vehicles composed of an ionizable lipid, cholesterol, phospholipids, and a polyethylene glycol (PEG)-lipid component that protect siRNA from enzymatic degradation, enhance systemic stability, facilitate intracellular delivery, and exhibit low toxicity and biodegradability (17–19). Clinical translation of this platform is demonstrated by Onpattro, the first FDA-approved RNA interference therapy for transthyretin-mediated amyloidosis, showing that systemic LNP-mediated siRNA delivery can achieve effective gene silencing in humans (17). These particles direct delivery to the liver. Once the formulation is delivered systemically, the modified PEG lipids dissociate and are replaced by adsorption of apolipoprotein E (ApoE) on the nanoparticle surface, which interacts with the cholesterol component of the particle to facilitate hepatic targeting (17). ApoE-coated LNPs serve as targeting ligands by binding to lipoprotein receptors on hepatocytes, triggering receptor-mediated endocytosis and efficient hepatic uptake (17). After internalization, the ionizable cationic lipid becomes protonated in the acidic endosomal environment, promoting endosomal escape through osmotic swelling and rupture, thereby releasing the siRNA into the cytoplasm (17). In the cytoplasm, siRNA is associated with the RNA-induced silencing complex (RISC), and during RISC assembly the double-stranded RNA is unwound so that the antisense strand remains bound to the complex (17). This antisense strand then guides sequence-specific binding and cleavage of target messenger RNA, ultimately preventing protein translation and gene expression (17).

In this study, porphyrin-lipid nanoparticles (porphyrin-LNPs) were formulated to encapsulate and deliver siRNA targeting CTNNB1 for the treatment of HCC. The porphyrin-LNP is based on the clinically approved Onpattro, which demonstrates the feasibility of systemic LNP-mediated RNA interference for gene silencing and therapeutic translation (17). Further modifications including porphyrin incorporation were introduced to evaluate the platform’s potential for treating HCC in this study. Porphyrin-LNP replaces the helper lipid DSPC found in Onpattro with porphyrin-lipid, which functions not only as a photosensitizer but also as a structural component that enhances intracellular siRNA delivery and gene silencing via light-activated siRNA endosomal release (LASER), as demonstrated in our previous study (20). The primary objective of this study is to validate the porphyrin-LNP platform as an effective siRNA delivery system targeting the undruggable target CTNNB1/β-catenin. This validation will provide a foundation for future studies leveraging its intrinsic photosensitizing property for potential clinical applications, including image-guided treatment planning and combination with light-controlled therapies (21).

## Materials and methods

### Genomic and clinical correlates of *CTNNB1* mutations in hepatocellular carcinoma

Publicly available HCC genomic datasets were interrogated to characterize the prevalence and clinical significance of *CTNNB1* alterations. Data were obtained from the four datasets including MSK 2023 (22), INSERM 2015 (23), the AMC 2014 (24), and the TCGA Firehose Legacy (25), comprising 943 HCC samples with available genomic profiling. *CTNNB1* alteration status was defined by reported somatic variants, with pathogenic mutations classified as *CTNNB1*-mutant. Mutation frequency was calculated as the proportion of *CTNNB1*-altered cases among profiled samples. Because activating mutations in exon 3 of *CTNNB1* disrupt β-catenin degradation and drive aberrant Wnt/β-catenin signaling, downstream analyses focused on samples harboring exon 3 mutations (n = 238) and compared them with *CTNNB1* wild-type controls (n = 705). Available clinical metadata was extracted according to data availability within each dataset. Tumor mutation burden was evaluated in 238 CTNNB1-mutant versus 655 wild-type cases, total mutation counts in 238 versus 657 cases, tumor size in 78 versus 148 cases, and age at diagnosis in 207 versus 612 cases. Group comparisons between *CTNNB1*-mutant and wild-type cases were performed using two-tailed unpaired Student’s t-tests.

### Formulation of porphyrin-LNP

Cholesterol, and 1,2-dimyristoyl-rac-glycero3-methoxy(poly(ethyleneglycol))-2000 (DMG-PEG-2000) were purchased from Avanti Polar Lipids (Alabaster, AL, USA). DLin-MC3-DMA and 1,2-Distearoyl-sn-glycero-3-phosphocholine (DSPC) were purchased from Nanosoft Polymers. Small Interfering RNAs (siRNAs) targeting CTNNB1 and scrambled control sequences were purchased from Horizon Discovery (USA). The sequences were as following: siCTNNB1 sense, 5’-GGUGGUGGUUAAUAAGGCU-3’ and siScramble sense, 5-UUCUCCGAACGUGUCACGU-3’. Porphyrin-lipid was synthesized using a previously reported microfluidic rapid mixing method (20). Porphyrin-LNPs were prepared using a previously reported microfluidic rapid mixing method (20). Briefly, lipids components were dissolved in ethanol at a molar ratio of 50/2/8/38.5/1.5 (DLin-MC3-DMA/porphyrin-lipid/DSPC/Cholesterol/DMG-PEG-2000). siRNAs were dissolved in 25 mM sodium acetate buffer (pH 4.0). The ethanol and aqueous phases were then mixed through herringbone-structured microfluidic chips (microfluidic ChipShop, Germany) at an aqueous-to-organic volume flow rate ratio of 3:1 and a total flow rate of 10 mL/min. The resulting solution was dialyzed overnight against phosphate-buffered saline (PBS, pH 7.4). Subsequently, the LNPs were concentrated using ultracentrifuge filters (Amicon, Sigma-Aldrich) and passed through a 0.22 μm filter before use.

### Characterization of porphyrin-LNP

The hydrodynamic diameter and polydispersity index of porphyrin-LNPs were measured using a Zetasizer Nano ZS (Malvern Instruments). Nanoparticle morphology was confirmed by transmission electron microscopy (TEM) and cryogenic electron microscopy (cryo-EM). The absorbance and fluorescence spectra of porphyrin-LNPs were acquired using a Cary 50 UV–vis spectrophotometer (Agilent, Mississauga, ON) and a FluoroMax fluorometer (Horiba Jobin Yvon, USA), respectively. The concentration and encapsulation efficiency of siRNA were quantified using the Quant-iT™ RiboGreen RNA Assay Kit (Thermo Fisher Scientific) according to the manufacturer’s instructions. Briefly, Porphyrin-lipid-siRNAs were diluted 1:1 in either Tris-EDTA (TE) buffer or 2% Triton X-100 in TE buffer. Following dilution, RiboGreen reagent was added to each well, and the mixtures were incubated at room temperature for 15 minutes. Fluorescence intensities were measured using a microplate reader (excitation/emission: 480/520 nm). Fluorescence signal from the TE buffer-treated samples (F_TE_) reflects the amount of unencapsulated siRNA, while signal from the Triton X-100-treated samples (F_TX_) represents total siRNA content. The encapsulation efficiency was calculated using the following equation:


$$\text{Encapsulation Efficiency}(\%)=\frac{F_{TX}-\;F_{TE}}{F_{TX}}X100\%$$

### Cryo-electron microscopy (cryo-EM) imaging of porphyrin-LNPs

To assess the intracellular delivery of siRNA, FAM-labeled siRNA (200 nM) was encapsulated within porphyrin-LNPs (~2 μM porphyrin) and incubated with Hep3B and HepG2 hepatocellular carcinoma (HCC) cells for 6 hours. The hydrodynamic size and poly dispersity of porphyrin-LNP were characterized using a Zetasizer Nano ZS (Malvern Instruments). The morphology of porphyrin-LNP was confirmed by cryo-EM image. Cryo-EM sample preparation and image acquisition: porphyrin-LNP-siRNA samples were concentrated to approximately 10–15 mg/mL of total lipid and used immediately for preparing cryo-grids. 4 μL of sample was deposited onto homemade holey gold grids glow-discharged at 15mA for 30 seconds prior to plunge-freezing into liquid ethane using an FEI Mark IV Vitrobot. Blotting conditions of 2 s blotting, +3 blot force, 5 °C ambient temperature, and 90% relative humidity were used. Cryo grids were loaded on a Glacios 200kV microscope (ThermoFisher) and datasets were collected with EPU (ThermoFisher) at a nominal magnification of 92,000× magnification (1.566 angstroms per pixel) at a target defocus range of 1–3 μm.

### Cell culture

The Hep3B (HB-8064) and HepG2 (HB-8065) human liver cancer cell lines were obtained from the American Type Culture Collection (ATCC) and tested negative for mycoplasma contamination. Hep3B and HepG2 cells were cultured in Dulbecco’s Modified Eagle Medium (DMEM) supplemented with 10% fetal bovine serum (FBS) and 1% penicillin–streptomycin in a humidified incubator at 37 °C with 5% CO_2_. All cell culture media and reagents were purchased from Life Technologies.

### Confocal imaging of intracellular uptake of porphyrin-LNPs

Cells were seeded into 8-well glass-bottom chambers (Nunc LabTek, Sigma-Aldrich, Rochester, NY) at a density of 2 × 10^4^ cells per well. After 48 hours, porphyrin-LNPs loaded with FAM-labeled siRNA were added at a concentration of 2 μM (based on porphyrin content) and incubated for 6 hours. Following incubation, cells were washed twice with culture medium to remove excess nanoparticles prior to imaging.

Confocal imaging was conducted at the Advanced Optical Microscopy Facility, University Health Network, using a Leica STED microscope (Leica Microsystems, Germany) equipped with a 63× oil immersion objective. Fluorescence signals were collected using customized filter settings: porphyrin-lipid was excited at 633 nm and emission was collected between 670–765 nm (laser power: 1–5%), while FAM-siRNA was excited at 488 nm and emission was collected between 507–580 nm (laser power: 30–60%).

### Real-time quantitative reverse transcription-polymerase chain reaction

Cells were seeded at a density of 1 × 10^5^ per well in a 6-well plate cultured with media and incubated overnight before treatment with porphyrin-LNPs at varying concentrations. After 24 hours, total RNA was extracted using RNeasy kits (QIAGEN) following the manufacturer’s protocol. Template cDNA for qPCR was synthesized from 1000 ng of total RNA that was extracted from transfected cells using iScript™ Reverse Transcription Supermix (BIO-RAD) according to the manufacturer’s protocol. The cDNA was diluted 1:10 using nuclease-free water, and 2 µL of the diluted cDNA was used to perform qPCR using PowerUp SYBR Green Master Mix containing qPCR master mix (Applied Biosystems) with specific primers (0.2 µM final concentration of each primer). Reactions were run on a StepOnePlus™ Real-Time PCR System (Applied Biosystems). Primer sequences were as follows: ACTB forward 5′-AGAGCTACGAGCTGCCTGAC-3′ and reverse 5′-AGCACTGTGTTGGCGTACAG -3′; GAPDH forward 5′-CACATCGCTCAGACACCATG-3′ and reverse 5′-GCAACAATATCCACTTTACCAGA -3′ and CTNNB1 forward 5′- CACAAGCAGAGTGCTGAAGGTG-3′ and reverse 5′-GATTCCTGAGAGTCCAAAGACAG-3′.

### Clonogenic survival assay

Cells were seeded at a density of 5,000 per well in a 6-well plate cultured with media and incubated overnight before being treated with porphyrin-LNP at varying concentrations. After 7 days, the cells were then washed twice with PBS, fixed with methanol and then stained with 0.1% crystal violet (Sigma-Aldrich). Subsequently, the plates were scanned for quantification using the ImageJ software.

### Pharmacokinetics assay of porphyrin-LNP

Blood samples were collected pre-injection from the femoral vein of 7-9-week-old male C57BL/6J mice (n = 5; The Jackson Laboratory) using Vaseline and heparinized capillary tubes. Post-injection of porphyrin-LNP (2 mg/kg via the lateral tail vein), blood samples were taken at specified intervals of 2 min, 5 min, 10 min, 15 min, 30 min, 1 hour, 2 hours, 4 hours, 8 hours, 24 hours, and 48 hours. The samples were then centrifuged to separate the plasma. Fluorescence emission measurements using CLARIOstar plus microplate reader (BMG Labtech) were performed after preparing 100X dilutions of the plasma samples with DMSO. The data were analyzed to create an exponential decay curve to determine the porphyrin-LNP’s plasma half-life.

### Generation of HCC mouse model

HCC mice models were generated using hydrodynamic tail vein injections of oncogene containing plasmids. Male C57BL/6J mice (The Jackson Laboratory) were uased in this HCC model. 20 μg of pT3-EF5α-G12D-mutant-K-Ras and pT3-EF5α-S45Ymutant-β-catenin-Myc-Tag (a kind gift from Dr. X. Chen at University of California, San Francisco) along with 6 μg PT2-C-Luc-PGK-SB13 (Plasmid #20207, Addgene) were diluted in 0.9% NaCl to a volume equivalent to 10% of the mouse body weight, and injected into the tail vein of 7-9-week-old C57BL/6J mice (The Jackson Laboratory) in 5–7 seconds. Two weeks after plasmid injection, the mice were randomly divided into three groups. Porphyrin-LNP-siRNA targeting CTNNB1 was administered intravenously at a dose of 2 mg/kg three times per week for three weeks (n = 13), with scrambled siRNA (n = 12) and PBS (n= 8) used as controls.

### Biodistribution assay of porphyrin-LNP

Porphyrin-LNP was intravenously injected at a dosage of 2 mg/kg in the male HCC mice (n = 3; The Jackson Laboratory). After 24-hours post-injection, *in vivo* and *ex-vivo* fluorescence imaging (excitation: 675 nm, emission: 720 nm) was performed using PerkinElmer Xenogen IVIS Spectrum Imaging System to detect the signal of porphyrin-LNP. Liver, kidneys, lungs, and heart were harvested and homogenized for fluorescence emission measurements of porphyrin-LNP using the CLARIOstar Plus microplate reader (BMG Labtech). The data were analyzed to calculate the percentage of the total injection dose of porphyrin-LNP per gram of tissue weight.

### Hematoxylin and eosin staining and tumor burden analysis

Liver tissues were harvested at the indicated time points, rinsed in PBS (Invitrogen), and fixed in 10% neutral-buffered formalin (Leica) for 24 hours at room temperature. Following fixation, tissues were processed through graded ethanol dehydration, cleared in xylene (Leica), and embedded in paraffin. Paraffin-embedded tissues were sectioned at 5 μm thickness using a microtome (Leica). Sections were deparaffinized in xylene (Leica), rehydrated through graded ethanol to distilled water, and stained with hematoxylin and eosin (Leica) according to standard protocols. Briefly, slides were stained with hematoxylin, rinsed, differentiated, blued, counterstained with eosin, dehydrated, cleared and coverslipped with permanent mounting medium. H&E slides were scanned using an Aperio AT2 scanner (Leica Biosystems) for tumor burden analysis. Tumor burden was assessed on whole-slide images based on established histopathological criteria and was calculated as the percentage of tumor area relative to total liver area. All histopathological evaluations were reviewed and performed by board-certified pathologists (Adeyi O) who were blinded to treatment allocation.

### Immunohistochemical analyses

Glass tissue section slides were dewaxed and rehydrated through a gradual decrease in ethanol concentration. The antigen epitopes on the tissue sections were unmasked using antigen retrieval buffer (Abcam, ab93678). The slides were then treated with 3% hydrogen peroxide to remove any endogenous peroxidases. Blocking was performed at room temperature with Dako protein block (Dako X0909) for 10 minutes. Sections were incubated at room temperature for 30 minutes with a primary myc-tag antibody at a 1:50 dilution (Cell Signaling Technology, 2278S). After primary incubation, sections were thoroughly washed with detergent phosphate-buffered saline TWEEN 20 (PBS-T) (PBS, Sigma-Aldrich, S0876; TWEEN 20, Sigma-Aldrich, P11379) and then incubated with a horseradish peroxidase-conjugated secondary antibody specific to the primary antibody used. After additional washes with PBS-T, the sections were treated with DAB substrate (Vector Laboratories, SK-4105) and incubated 10 minutes for signal development before stopping the reaction in water. Finally, the sections were lightly counterstained with hematoxylin, dehydrated through a gradual increase in ethanol concentration, cleared in xylene, and mounted in Permount (Thermo Fisher Scientific). Subsequently, the IHC slides were scanned by Aperio AT2 (Leica Biosystems) for quantification using the positive pixel count algorithm in Aperio ImageScope (Leica Biosystems).

### Statistical analyses

Values are given as mean ± SD. Statistical significance was assessed using the two-tailed unpaired Student’s t test or one-way ANOVA test with *p* values (Prism Software). A *p*-value < 0.05 was considered statistically significant.

## Results

### CTNNB1 mutations are associated with poor prognostic features and reduced therapeutic response in HCC

**Figure 1:**
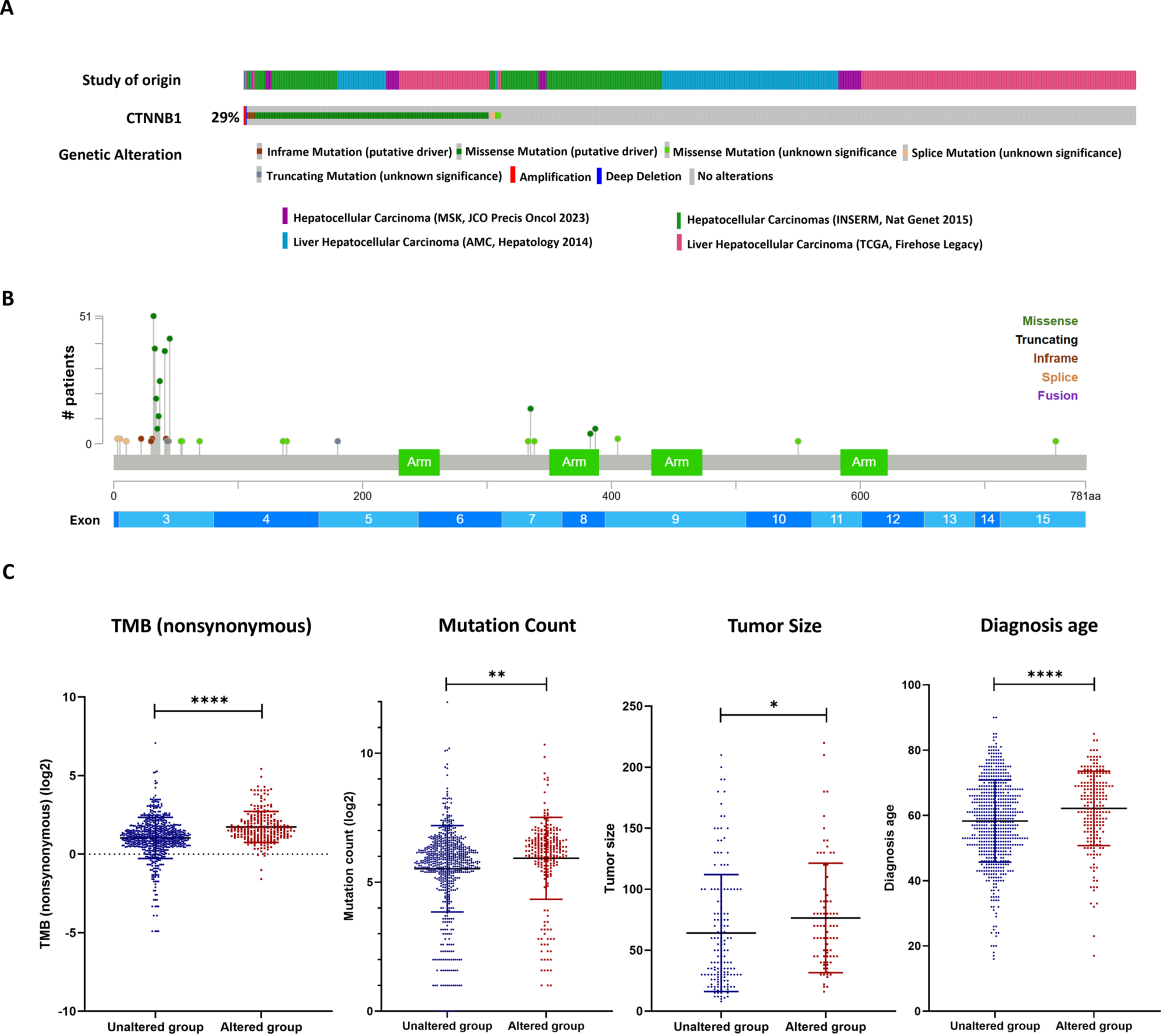
CTNNB1 alterations correlate with clinical parameters. **(A)** Oncoprint plot showing the frequency and types of *CTNNB1* genetic alterations across multiple HCC cohorts. **(B)** Lollipop plot showing the distribution and frequency of somatic *CTNNB1* mutations across *CTNNB1* exons in HCC. **(C)** Clinical correlations comparing exon 3 *CTNNB1*-mutant and wild-type HCC patients across four parameters: tumor mutation burden (TMB), total mutation count, tumor size, and age at diagnosis. *p < 0.05, **p < 0.01, ****p < 0.0001, two-tailed unpaired Student’s t test.

Analysis of multiple available HCC datasets, including MSK 2023 (22), INSERM 2015 (23), AMC 2014 (24), and TCGA Firehose Legacy (25), revealed that *CTNNB1* was altered in 29% of profiled patients (260 out of 902 cases), yielding 275 *CTNNB1*-mutant samples among 943 total samples (**Figure 1A**). Given that *CTNNB1* mutations predominantly occur in exon 3, which prevents degradation of β-catenin and results in aberrant activation of the Wnt/β-catenin signaling pathway (26), 238 samples with exon 3 *CTNNB1* mutations were included for downstream analyses (**Figure 1B**). Comparison of clinical features between exon 3 *CTNNB1*-mutant and *CTNNB1*-wild-type HCC cases showed that *CTNNB1* mutations were associated with increased tumor mutation burden (TMB) (mean: 4.42 vs. 3.09 mutations/Mb, *p* < 0.0001) and an elevation in total mutation count (mean: 96.83 vs. 81.36, *p* = 0.0012) (**Figure 1C**). In addition, tumor size was larger in exon 3 *CTNNB1*-mutant cases (mean: 76.4 mm) compared to wild-type tumors (mean: 64.1 mm, *p* = 0.0303), and age at diagnosis was higher in the *CTNNB1*-mutant group (mean: 62.15 vs. 58.28 years p < 0.0001) (**Figure 1C**). Higher TMB, mutation count and tumor size were reported to be associated with worse survival outcomes in HCC and resistance to tyrosine Kinase Inhibitors (TKI) monotherapy (27–29).

### Porphyrin-LNP characterization and siRNA delivery in HCC cell lines

**Figure 2:**
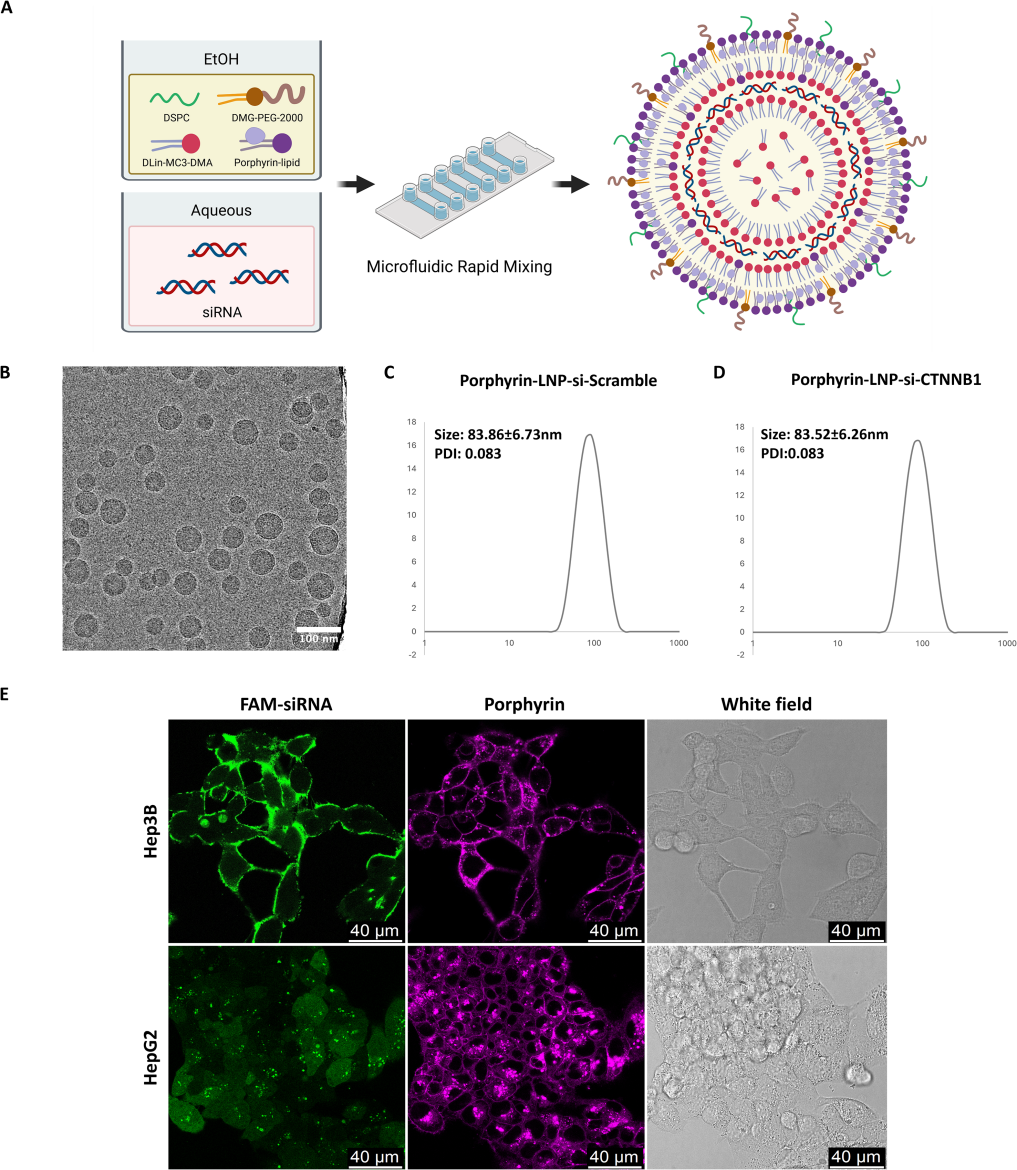
Porphyrin-LNP synthesis and characterization. **(A)** Porphyrin-LNPs were synthesized via microfluidic rapid mixing. **(B)**. Cryo-TEM image of porphyrin-LNP (scale bar = 100 nm). **(C)** Size distribution of porphyrin-LNP-si-Scramble and **(D)** porphyrin-LNP-si-CTNNB1 measured by dynamic light scattering (DLS). **(E)** Confocal imaging of FAM-siRNA delivery by porphyrin-LNP. Hep3B and HepG2 cells were treated with porphyrin-LNP encapsulating FAM-siRNA (200 nM, ~2 μM porphyrin) for 6 h. Confocal images show FAM-siRNA (green) and porphyrin (magenta).

Porphyrin-LNPs were synthesized using a standard rapid mixing method through a microfluidic system (**Figure 2A**), as described in the Methods section. Cryo-TEM revealed that the resulting porphyrin-LNPs displayed uniform, spherical amorphous core structures (**Figure 2B**). Dynamic light scattering (DLS) analysis showed that the average hydrodynamic diameters of porphyrin-LNPs encapsulating scrambled siRNA and CTNNB1-targeting siRNA were 83.86 ± 6.73 nm with polydispersity index (PDI) < 0.1 and 83.52 ± 6.26 nm with PDI <0.1, respectively (**Figures 2C, D**). Both formulations demonstrated high encapsulation efficiencies, with 97.5% for si-Scramble and 97.0% for si-CTNNB1. Confocal fluorescence microscopy was then employed to visualize siRNA uptake and intracellular localization (green: FAM-siRNA; magenta: porphyrin) (**Figure 2E**). Efficient cellular uptake of FAM-siRNA was observed in both HCC cell lines. Notably, FAM-siRNA was predominantly localized near the cell membrane in Hep3B cells, whereas it was broadly distributed in the cytosol in HepG2 cells, suggesting that intracellular siRNA release may vary in a cell type–dependent manner.

### Downregulation of *CTNNB1* and reduced clonogenic survival using porphyrin-LNP-siRNA in HCC cell lines

**Figure 3:**
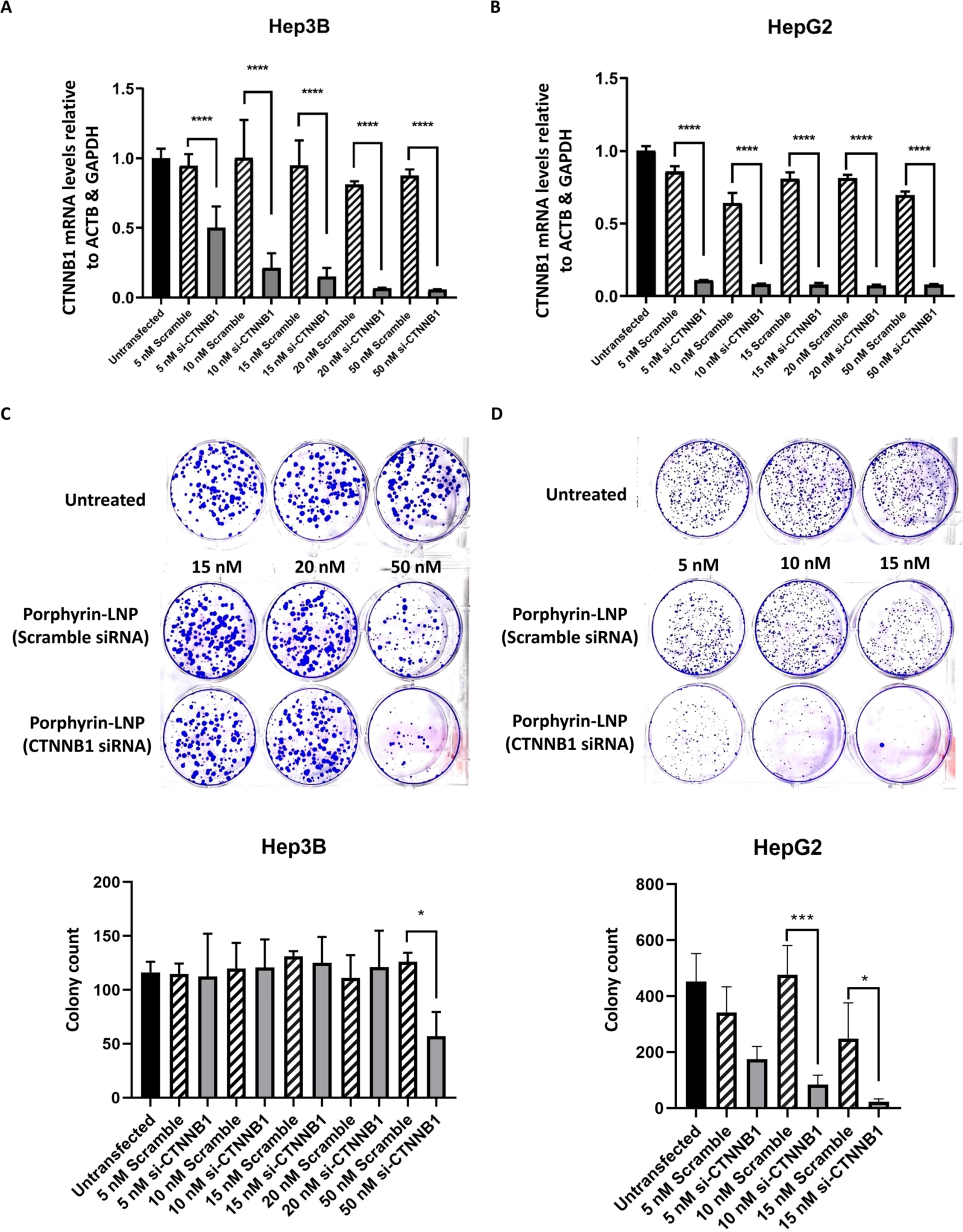
Validation of *CTNNB1* knockdown efficiency using porphyrin-LNP-siRNA. Downregulation of *CTNNB1* expression after transfection of porphyrin-LNP-siRNA targeting *CTNNB1* in **(A)** Hep3B and **(B)** HepG2 cells. ****p < 0.0001; one-way ANOVA test. Reduced colony formation after transfection of porphyrin-LNP-siRNA targeting *CTNNB1* in **(C)** Hep3B and **(D)** HepG2 cells. *p < 0.05, ***p < 0.001, one-way ANOVA test.

Porphyrin-LNP-siRNA was transfected into Hep3B and HepG2 HCC cells, both with active Wnt/β-catenin signaling, to confirm its effectiveness in downregulating *CTNNB1*. Hep3B and HepG2 were used in this study because Hep3B cells possess non-mutated β-catenin protein with active Wnt/β-catenin signaling, whereas HepG2 cells harbor an exon 3–4 truncation mutation in the *CTNNB1* gene (30, 31). This mutation results in the aberrant accumulation of β-catenin, leading to the constitutive activation of the Wnt/β-catenin pathway (30, 31). Importantly, porphyrin-LNP-siRNA targeting *CTNNB1* was confirmed to downregulate *CTNNB1* expression in both cell lines after 24 hours of transfection compared with scramble controls, achieving up to 90% knockdown in Hep3B cells at 50 nM (*p* < 0.0001) and 90% knockdown in HepG2 cells at 10 nM (*p* < 0.0001). (**Figures 3A, B**). The clonogenic survival assay was performed to further examine the phenotypic effects after transfection in both cell lines. Clonogenic survival was significantly reduced following porphyrin-LNP-siRNA treatment targeting *CTNNB1*, with a 50% reduction in Hep3B at 50 nM (*p* < 0.05) and reductions of 75% (*p* < 0.001) and 90% (*p* < 0.05) in HepG2 at 10 nM and 15 nM, respectively (**Figures 3C, D**). These results validate that the porphyrin-LNP-siRNA is functional in downregulating *CTNNB1* expression and reducing clonogenic survival in the HCC cell line in the background of active Wnt/β-catenin signaling and mutant *CTNNB1*.

### Pharmacokinetics and biodistribution of porphyrin-LNP in HCC mouse models

**Figure 4:**
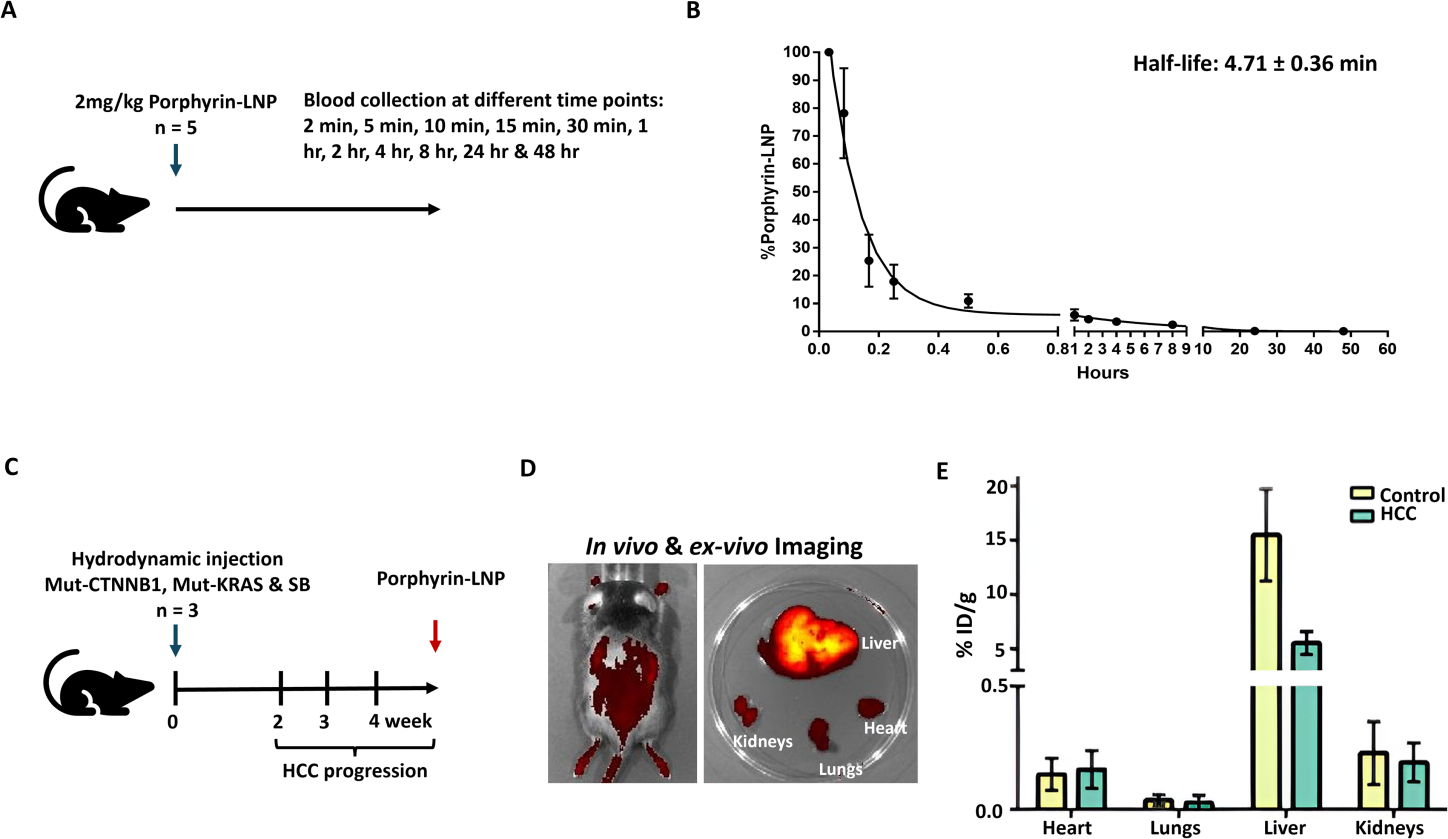
Pharmacokinetics and biodistribution of Porphyrin-LNP-siRNA **(A)** Experimental design for plasma clearance analysis. C57BL/6J mice (n = 5) received a single intravenous injection of porphyrin-LNP (2 mg/kg), and blood samples were collected at 2, 5, 10, 15, and 30 minutes, and 1, 2, 4, 8, 24, and 48 hours post-injection. **(B)** Experimental design for plasma clearance analyPlasma clearance analysis showed a half-life of 4.71 ± 0.36 minutes, and porphyrin-LNP was undetectable in plasma 24 hours post-injection. **(C)** Experimental scheme for biodistribution analysis in the hydrodynamic injection–induced HCC mouse model (n = 3). Porphyrin-LNP was administered intravenously four weeks after tumor induction. **(D)** Representative *in vivo* and *ex vivo* fluorescence imaging 24 hours after intravenous injection of porphyrin-LNP. Strong fluorescent signal (λ_em = 720 nm) was observed in the liver compared to other harvested organs (heart, lungs, kidneys). **(E)** Quantitative biodistribution analysis expressed as percentage of injected dose per gram of tissue (%ID/g) 24 hours post-injection, demonstrating preferential accumulation of porphyrin-LNP in HCC livers (5% ID/g) compared to other organs.

Blood clearance of porphyrin-LNP was evaluated following a single intravenous injection (2 mg/kg) in C57BL/6J mice (n = 5) (**Figure 4A**). Pharmacokinetic analysis revealed a rapid plasma clearance profile, with a calculated half-life of 4.71 ± 0.36 minutes (**Figure 4B**). Porphyrin-LNP levels declined sharply within the first 30 minutes and were undetectable in plasma by 24 hours post-injection, indicating efficient systemic clearance (**Figure 4B**). Given that porphyrin-LNP emits a near-infrared fluorescent signal (λ_em = 720 nm), whole-body *in vivo* and *ex vivo* organ fluorescence analysis was performed to assess tissue distribution in the hydrodynamic injection–induced HCC mouse model (n = 3) (**Figures 4C, D**). At 24 hours post-injection, strong fluorescence signals were observed predominantly in the liver, with comparatively lower signal intensity detected in the heart, lungs, and kidneys (**Figure 4D**). Quantitative biodistribution analysis demonstrated preferential hepatic accumulation, reaching approximately 5% of the injected dose per gram of tissue (%ID/g) in HCC livers (**Figure 4E**). These findings indicate that despite rapid plasma clearance, porphyrin-LNP efficiently accumulates in liver tumor tissue, supporting its suitability for liver-targeted RNA delivery applications.

### Anti-tumor therapeutic efficacy using porphyrin-LNP-siRNA targeting *CTNNB1* in HCC mouse model

**Figure 5:**
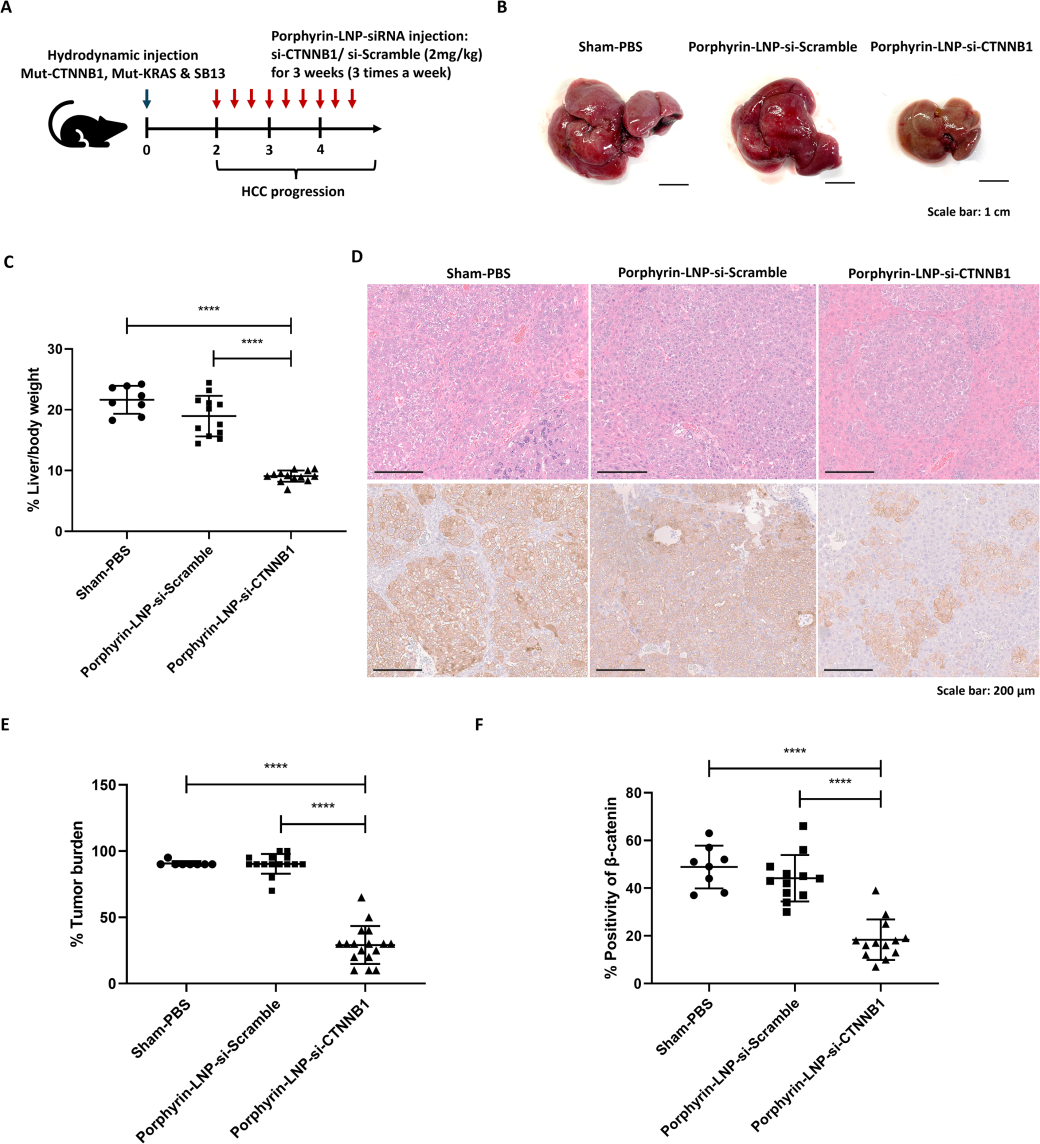
Porphyrin-LNP-siRNA targeting *CTNNB1* for HCC treatment. **(A)** The protocol describes the treatment of HCC mouse model using porphyrin-LNP-siRNA targeting *CTNNB1*. Two weeks following plasmid injection for tumor induction, porphyrin-LNP-siRNA was administered intravenously at a dose of 2 mg/kg three times per week for a total duration of three weeks. Experimental cohorts included Sham-PBS (n = 8), scramble siRNA-LNP (n = 12), and *CTNNB1* siRNA-LNP (n = 13). **(B)** Gross liver appearance of mice treated with porphyrin-LAN-siRNA targeting *CTNNB1*, scramble and sham control. **(C)** Liver-to-body weight ratio of mice treated with porphyrin-LAN-siRNA targeting *CTNNB1* compared to scramble and sham control. **(D)** Representing H&E images (top) and CTNNB1 IHC staining (bottom) of mice treated with porphyrin-LAN-siRNA targeting *CTNNB1*, scramble and sham control (20× magnification). **(E)** Semi-quantitative analysis of tumor grading of mice treated with porphyrin-LAN-siRNA targeting *CTNNB1*, scramble and sham control. **(F)** Quantitative analysis of CTNNB1 IHC staining in mice treated with porphyrin-LAN-siRNA targeting *CTNNB1*, scramble and sham control. ****p < 0.0001, one-way ANOVA test.

Murine HCCs were generated using hydrodynamic transfection to overexpress mutant CTNNB1 and mutant KRAS in livers. Two weeks after plasmid injection, porphyrin-LNP-siRNA targeting CTNNB1 was administered intravenously at a dose of 2 mg/kg three times a week for three weeks (n = 13) (**Figure 5A**). Scramble siRNA (n= 12) and a sham group (n = 8) receiving PBS injection served as controls. Importantly, mice with HCC treated with porphyrin-LNP-siRNA targeting CTNNB1 exhibited a significantly lower liver-to-body weight ratio compared to the scramble control and sham group corresponding to an approximately 50% reduction (*p* < 0.0001) (**Figures 5B, C**). The representative H&E images of mice treated with porphyrin-LNP-siRNA targeting CTNNB1 and all control groups were shown (**Figure 5D**), and histopathological analysis by a histopathologist scored significantly lower tumor grading, corresponding to an approximately 67% reduction compared to all control groups (*p* < 0.0001) (**Figure 5E**). IHC staining showed a significant decrease in β-catenin protein expression in mice treated with porphyrin-LNP-siRNA targeting CTNNB1, corresponding to an approximately 58% reduction (*p* < 0.0001) compared to all controls (**Figures 5D, F**). Collectively, these findings demonstrated that porphyrin-LNP-mediated delivery of CTNNB1 siRNA suppressed tumor burden and oncogenic signaling in the HCC model, highlighting its therapeutic potential for targeting β-catenin–driven HCC.

## Discussion

In this study, we demonstrate that systemic delivery of porphyrin-LNP carrying siRNA against CTNNB1 significantly inhibits tumor burden in a mouse HCC model driven by oncogenic CTNNB1 (S45Y) and K-Ras (G12D) mutations. HCC mice treated with this formulation exhibited a significant reduction in liver-to-body weight ratio, lower tumor grading, and substantial downregulation of β-catenin protein levels. These findings collectively suggest that our porphyrin-LNP-siRNA platform effectively inhibits mutant CTNNB1/KRAS-driven hepatocarcinogenesis.

S45 mutations of CTNNB1 are detected in approximately 20% of mutated Hepatocellular Adenomas (HCAs) and HCCs and are generally classified as weak β-catenin activators compared with other CTNNB1 hotspot mutations (26). Notably, many HCCs harboring S45 mutations exhibit duplication of the mutant allele, resulting in high overall β-catenin activity despite the intrinsically weaker signaling output of a single S45-mutant allele (26). Although co-delivery of a transposon plasmid encoding CTNNB1-S45Y together with a transposase supports genomic integration analogous to allele gain, the hydrodynamic tail vein injection approach still produces diffuse and widespread β-catenin activation throughout the liver parenchyma. This pattern does not fully recapitulate the focal tumorigenesis observed in human HCC. More physiologically relevant models, such as hepatocyte-specific CTNNB1 exon 3 deletion or Apc frameshift (Apc^fs-ex15) mouse models, have been shown to generate tumors that are phenotypically and transcriptionally similar to well-differentiated CTNNB1-mutated human HCCs (32). Furthermore, recent work demonstrates that CRISPR/Cas9-mediated exon skipping of Ctnnb1 can generate gain-of-function β-catenin isoforms and produce tumor subtypes that closely reflect the histological and transcriptional heterogeneity of human liver cancers (33). Incorporation of these alternative models in future studies will be critical for determining whether our porphyrin-LNP–siRNA platform maintains therapeutic efficacy across the full spectrum of β-catenin–driven hepatocarcinogenesis.

To date, therapeutic strategies targeting CTNNB1 have been unsuccessful. Small-molecule inhibitors either lack specificity or fail to achieve sufficient tumor penetration, and conventional siRNA approaches have suffered from poor delivery efficiency, rapid degradation, or hepatic off-target effects (34). Currently, there are currently no approved therapies that directly target CTNNB1, and clinical strategies aimed at this pathway have yielded limited success. Only one clinical trial (NCT06600321) is investigating ALN-BCAT, a lipid nanoparticle-formulated, chemically modified siRNA specifically targeting β-catenin. Most ongoing efforts focus on targeting downstream effectors or coactivators of CTNNB1. For example, current clinical trials are investigating agents such as DKN-01, a monoclonal antibody against DKK1 (NCT03645980); Tegavivint, which disrupts β-catenin binding to the TBL1 coactivator (NCT04851119); and meclizine, a constitutive androstane receptor (CAR) inverse agonist that indirectly modulate Wnt signaling (NCT03253289). Parallel efforts have explored nanoparticle-based formulations targeting other components of the Wnt pathway, such as MYC-targeted siRNA or mRNA lipid nanoparticles including DCR-MYC (NCT02314052) and OTX-2002 (NCT05497453), as well as small activating RNAs like MTL-CEBPA that target C/EBP-α (NCT02716012); however, none of these approaches directly silence CTNNB1.

Our study addresses these unmet needs by employing porphyrin-LNPs to deliver siRNA specifically against CTNNB1. Moreover, porphyrin-LNPs have the potential to deliver siRNAs to cancer cells while offering possibilities for activatable fluorescence imaging, photodynamic therapy, ^64^Cu radiolabeling and image guidance for treatment dosage titration (35, 36). Porphyrin-based LNP systems have previously been investigated across multiple oncologic contexts. Prior studies have applied porphyrin-grafted LNPs for photodynamic therapy and ferroptosis induction in colorectal cancer models (37), oxygen self-supplemented PDT for liver metastasis of colon cancer (38), structural modulation of ApoE-mediated uptake to enhance intracellular delivery (39), and light-activated siRNA endosomal release (LASER) strategies in prostate cancer (20). These investigations primarily leveraged the photophysical properties of porphyrins for imaging, reactive oxygen species generation, or endosomal membrane disruption. Various LNP-based RNA delivery platforms have also been explored targeting genes such as PLK1, Jnk2, SALL4, YTHDF1, VEGFR2, Midkine, BCL-2, IGF1R, MEF2D, and DNAJB1-PRKACA, using strategies including ApoE coating, GalNAc conjugation, peptide modification, macrophage membrane camouflage, HDL-like particles, and ultra-small or thermosensitive formulations in HCC (40–51). In contrast to these prior approaches, our study focuses specifically on systemic siRNA-mediated targeting of oncogenic CTNNB1 using a porphyrin-based LNP platform in a genetically driven HCC model, enabling direct silencing of an undruggable Wnt pathway driver. This approach may contribute to addressing key delivery challenges that have limited Wnt pathway–targeted therapies in HCC. Further investigation is required to evaluate long-term efficacy, safety, and translational potential.

To date, only two published studies have reported the use of LNPs to deliver siRNA targeting CTNNB1 in HCC, highlighting the emerging feasibility of RNA interference–based strategies for β-catenin–driven tumors (15, 16). Importantly, these studies evaluated LNP-mediated CTNNB1 silencing in both orthotopic patient-derived xenograft (PDX) models and CTNNB1-driven hydrodynamic transfection HCC models, demonstrating significant tumor suppression across complementary experimental systems. In those studies, treatment resulted in approximately 70% reduction in tumor burden and 60% reduction in β-catenin protein expression (15, 16), which is comparable to the therapeutic response observed in our CTNNB1-driven hydrodynamic transfection HCC model following treatment with porphyrin-LNP–encapsulated siRNA targeting CTNNB1. However, several limitations should be acknowledged. Unlike prior studies that incorporated orthotopic PDX models, our work utilized a hydrodynamic transfection–induced murine HCC model driven by mutant CTNNB1 and KRAS. While this model recapitulates key oncogenic signaling features, it does not fully capture the molecular heterogeneity and tumor microenvironment complexity of human HCC. Future studies incorporating orthotopic and PDX models will be essential to further validate therapeutic efficacy and assess translational potential in more clinically relevant settings. Furthermore, previous studies have demonstrated that CTNNB1 inhibition using LNPs is associated with reduced expression of canonical Wnt target genes, such as *Glul*, *Ccnd1*, *Lect2*, and *Rgn*, as well as a shift toward a more pro-inflammatory immune landscape, including increased infiltration of M1-like macrophages (16). While our study demonstrates effective β-catenin knockdown and antitumor efficacy, the lack of comprehensive profiling of downstream transcriptional and immune changes represents a limitation of the present work. Future studies incorporating systematic approaches such as single-cell RNA sequencing will be important for further delineating the molecular and immune landscapes associated with CTNNB1 silencing *in vivo*. In addition, β-catenin rescue experiments would provide further mechanistic validation of CTNNB1-dependent effects and represent an important direction for future investigation.

In conclusion, our data highlight the potential of porphyrin-LNP-mediated siRNA delivery to overcome longstanding barriers in HCC treatment. By enabling effective silencing of an undruggable oncogene, this strategy provides a strong foundation for further translational development. Our data demonstrated prolonged circulation and tumor accumulation of porphyrin-LNPs following intravenous administration; however, the absence of comparative administration routes, such as intraperitoneal or subcutaneous, limits conclusions regarding route-dependent pharmacokinetics. Future studies incorporating multiple delivery routes will be necessary to delineate optimal administration strategies and translational potential. Moreover, future studies should investigate intrahepatic biodistribution, immune modulation, and combination regimens in clinically relevant settings to fully realize the therapeutic potential of CTNNB1-targeted RNA interference in liver cancer.

## Data Availability

The datasets presented in this study can be found in online repositories. The names of the repository/repositories and accession number(s) can be found in the article/supplementary material.
